# Two New Reported Species of *Longidorus* spp. and *Xenocriconemella* spp. from Mainland Greece

**DOI:** 10.2478/jofnem-2025-0050

**Published:** 2026-01-14

**Authors:** Ana García Velázquez, Dionysios Ntinokas, Carolina Cantalapiedra-Navarrete, Ioannis Giannakou, Juan E. Palomares-Rius, Emmanuel A. Tzortzakakis, Pablo Castillo, Antonio Archidona-Yuste

**Affiliations:** Institute for Sustainable Agriculture (IAS), Department of Crop Protection, CSIC, 14004 Córdoba, Spain; Laboratory of Agricultural Zoology and Entomology, Department of Science of Crop Production, Agricultural University of Athens, 11855 Athens, Greece; Institute of Olive Tree, Subtropical Crops and Viticulture, Department of Viticulture, Vegetable Crops, Floriculture and Plant Protection, ELGO-DIMITRA, 71307 Heraklion, Crete, Greece

**Keywords:** cytochrome *c* oxidase subunit 1, D2–D3 of 28S rDNA, detection, ITS rDNA, needle and ring nematodes

## Abstract

Nematode sampling was conducted to identify ring and needle nematodes in forests of central and northern Greece. Two species of the genus *Xenocriconemella*, *X. iberica* and *X. paraiberica*, and three species of *Longidorus, L. aetnaeus, L. intermedius*, and *L. iranicus*, were identified based on integrative taxonomy. To our knowledge, this is the first report of *X. iberica* and *X. paraiberica* in Greece, and the first time these species have been documented outside the Iberian Peninsula. Similarly, *L. aetnaeus* and *L. intermedius* are reported from Greece for the first time. This study expands the known geographic distribution of *Xenocriconemella* and *Longidorus* species in Greece and the broader Mediterranean Basin. Molecular characterization confirms that two morphologically distinct species, *L. intermedius* and *L. piceicola*, despite differences in lip region width, odontostyle and body lengths, exhibit high similarity in the D2–D3 expansion segments of 28S and internal transcribed spacer 1 (ITS1) rDNA regions. This close molecular affinity underscores the need for further investigation using additional nuclear (e.g., *hsp90*) and mitochondrial (e.g., *COI*) markers to clarify the extent of genetic divergence between these two needle nematode species.

## Introduction

Needle and ring nematodes, belonging to the genera *Longidorus* Micoletzky, 1922, and *Xenocriconemella* De Grisse and Loof, 1965, are polyphagous root ectoparasites that feed directly on root cells of a wide range of wild and economically important plants (Jairajpuri and Ahmad, 1992; Geraert, 2010). To date, no *Xenocriconemella* species have been reported from Greece, while 15 *Longidorus* species have been documented in the country. However, only 10 of these have been identified using molecular methods: *L. closelongatus* Stoyanov, 1964; *L. cretensis* Tzortzakakis, Peneva, Terzakis, Neilson and Brown, 2001; *L. euonymus* Mali and Hooper, 1974; *L. iranicus* (=*moesicus*) Sturhan and Barooti, 1983; *L. kuiperi* Brinkman, Loof and Barbez, 1987; *L. leptocephalus* Hooper, 1961; *L. orientalis* Loof, 1982; *L. pisi* (=*latocephalus*) Edward, Misra and Singh, 1964; *L. pseudoelongatus* Altherr, 1976; and *L. pauli* Lamberti, Molinari, De Luca, Agostinelli and Di Vito, 1999 (He et al., 2005; Tzortzakakis et al., 2014, 2016, 2017, 2021; Clavero-Camacho et al., 2022). For the remaining 5 species, *L. africanus* Merny, 1966; *L. elongatus* (de Man, 1876) Micoletzky, 1922; *L. fasciatus* Roca and Lamberti, 1981; *L. intermedius* Kozlowska and Seinhorst, 1979; and *L. proximus* Sturhan and Argo, 1983, no molecular data are currently available (Tzortzakakis et al., 2008).

Given the large number of species in the genus *Longidorus* (194 nominal species, Monemi et al., 2024; Jahanshahi Afshar, 2025; Salazar-García et al., 2025) and the limited morphological variability within *Xenocriconemella*, including frequent detection of only juvenile stages in soil samples, DNA barcoding techniques have increasingly been employed using nuclear ribosomal and mitochondrial markers for accurate species identification. Advances in molecular taxonomy for both needle and ring nematodes now provide powerful tools for *Longidorus* and *Xenocriconemella* identification (Archidona-Yuste et al., 2016, 2019, 2024). Integrating multiple genetic markers, particularly the D2–D3 expansion segments of 28S rDNA, the internal transcribed spacer (ITS) region, and the cytochrome *c* oxidase subunit I (*COI*) gene with morphometric species delimitation, has proven highly effective in resolving species complexes within these genera.

During a recent nematode survey conducted in forested areas dominated by downy oak (*Quercus pubescens* Willd.) across central and northern mainland Greece (Thessaloniki, Pieria, Tatoi, and Chalkidiki), four populations of ring nematodes and four populations of needle nematodes, belonging to *Xenocriconemella* and *Longidorus*, were detected. The objective of this study was to accurately identify these nematode population through a combination of morphological characterization and molecular analyses, including sequencing of the D2–D3 expansion segments of 28S rDNA, the ITS1 region, and partial mitochondrial *COI* genes.

## Materials and Methods

### Nematode samples and morphological study

Soil samples containing needle (*Longidorus* spp.) and ring nematodes (resembling *Xenocriconemella* spp.) were collected using a metallic sampler (internal diameter 2.5 cm) from the upper 30 cm of soil beneath three to four randomly selected downy oak trees at each of four forest sites: Thessaloniki (40°42′62.15″ N, 23°50′18.52″ E), Pieria (40°14′24.01″N, 22°10′48.01″E), Chalkidiki (40°25′34.40″ N, 23°30′6.70″ E), and Tatoi (38°07′19.20″ N, 23°49′58.79″ E), located in northern and central regions of mainland Greece, respectively. From each bulk soil sample, a 500 cm^3^ subsample was processed for nematode extraction via centrifugal flotation (Coolen, 1979). Extracted specimens were heat-killed, fixed in a solution of 4% formaldehyde and 1% propionic acid, and subsequently processed into pure glycerin following Seinhorst’s (1966) method. Morphometric measurements and light micrographs, including de Man indices, body length, odontostyle length, lip region width, tail length and shape, guiding ring distance from the anterior end, and body annuli, were obtained using a Leica DM6 compound microscope equipped with a Leica DFC7000 T digital camera. Terminology, ratios, and abbreviations follow those defined by Jairajpuri and Ahmad (1992) and Siddiqi (2000).

### Molecular characterization

DNA was extracted from individual needle and ring nematodes, and PCR assays were performed as previously described, targeting the D2–D3 expansion segments of 28S rDNA, ITS rDNA, and partial mitochondrial *COI* regions. DNA extractions and PCR assays were conducted following the protocol of Archidona-Yuste et al. (2016, 2024). Amplification of the D2–D3 expansion segments of the 28S rDNA was performed using primers D2Ab (5′-ACAAGTACCGTGAGGGAAAGTTG-3′) and D3B (5′-TCGGAAGGAACCAGCTACTA-3′) (De Ley et al., 1999). For needle nematode populations, the ITS1 located between the 18S and 5.8S rDNA was amplified using forward primer 18S (5′-TTGATTACGTCCCTGCCCTTT-3′) (Vrain et al., 1992) and the reverse primer rDNA1 5.8S (5′-ACGAGCCGAGTGATCCACCG-3′) (Cherry et al., 1997). For ring nematode populations, ITS amplification employed forward primer TW81 (5′-GTTTCCGTAGGTGAACCTGC-3′) and reverse primer AB28 (5′-ATATGCTTAAGTTCAGCGGGT-3′) (Subbotin et al., 2001). Additionally, a fragment of the mitochondrial cytochrome *c* oxidase I (*COI*) gene in needle nematodes was amplified following Lazarova et al. (2006), using primers COIF (5′-GATTTTTTGGKCATCCWGARG-3′) and COIR (5′-CWACATAATAAGTATCATG-3′) (Hu et al., 2002; Derycke et al., 2005). PCR conditions for all reactions followed Archidona-Yuste et al. (2016, 2024). Amplicons were purified using ExoSAP-IT (Affymetrix, USB Products) and directly sequenced on a 3130XL Genetic Analyzer (Applied Biosystems, Foster City, CA, USA) using the BigDye Terminator v3.1 Cycle Sequencing Kit (Applied Biosystems). Sequencing was performed at Stab Vida (Caparica, Portugal). Chromatograms for all three markers (D2–D3 expansion segments of 28S rDNA, ITS rDNA, and *COI*) were analyzed using DNASTAR Lasergene SeqMan v.7.1.0. Species identity of the obtained sequences was confirmed using the basic local alignment search tool (BLAST) hosted by the National Center for Biotechnology Information (NCBI) (Altschul et al., 1990). Newly generated sequences were deposited in NCBI under the accession numbers listed in Table 1 and the phylogenetic trees.

**Table 1:** Needle and ring nematode populations belonging to the genera *Longidorus* and *Xenocriconemella*, collected from downy oak (*Quercus pubescens* Willd.) forests in Greece, were used and sequenced in this study.

**Species**	**Sample code**	**Location**	**D2–D3**	**ITS1/ITS**	***COI***
*Longidorus aetnaeus*	THE2	Thessaloniki, Northern Greece	PV917559–PV917560	PV891811–PV891812	PV871896–PV871897
*Longidorus intermedius*	THE1	Thessaloniki, Northern Greece	PV917561–PV917566	PV891813–PV891816	PV871898–PV871901
*Longidorus intermedius*	PIE2	Pieria, Northern Greece	PV917567–PV917572	PV891817–PV891819	PV871902–PV871905
*Longidorus iranicus*	T098	Tatoi, Central Greece	PV917573	-	-
*Xenocriconemella iberica*	THE2	Thessaloniki, Northern Greece	PV917574–PV917578	PV891820–PV891824	-
*Xenocriconemella paraiberica*	THE2	Thessaloniki, Northern Greece	PV917579–PV917583	PV891825–PV891829	-
*Xenocriconemella paraiberica*	PIE6	Pieria, Northern Greece	PV917584–PV917588	PV891830–PV891834	-
*Xenocriconemella paraiberica*	T106	Chalkidiki, Northern Greece	PV917589–PV917590	PV891835–PV891836	-

-Not sequenced.

ITS, internal transcribed spacer.

### Phylogenetic analyses

Phylogenetic analyses were conducted exclusively on *Longidorus* nematode populations based on the D2–D3 expansion segments of 28S rDNA, ITS1 rDNA, and *COI* mtDNA sequences. This focus was due to the availability of newly generated ITS1 sequences for *L. aetnaeus* Roca, Lamberti, Agostinelli, and Vinciguerra, 1986. By contrast, further analysis of *Xenocriconemella* sequences was deemed unnecessary, as their D2–D3 and ITS rRNA regions exhibited high identity to sequences already deposited in NCBI. These sequences, along with additional *Longidorus* sequences retrieved from NCBI, were used in the analyses. Outgroup taxa for each dataset were selected based on previous studies (Archidona-Yuste et al., 2016; Cai et al., 2020; Liébanas et al., 2022).

Multiple sequence alignments were performed for each gene using the FFT-NS-2 algorithm in MAFFT v7.450 (Katoh et al., 2019). Alignments were visualized with BioEdit v7.2.5 (Hall, 1999) and manually curated to remove poorly aligned regions. A light filtering strategy, eliminating up to 20% of alignment positions, was applied, following the recommendations of Tan et al. (2015), to enhance phylogenetic accuracy and reduce computational time. This approach was favored over automated filtering methods, which have been shown to compromise single-gene phylogenetic inference (Tan et al., 2015). Bayesian inference (BI) analyses were carried out using MrBayes v3.1.2 (Ronquist and Huelsenbeck, 2003). The optimal models of DNA evolution were selected using JModelTest v2.1.7 (Darriba et al., 2012) based on the Akaike information Criterion (AIC). The selected models, including base frequencies, proportions of invariable sites, gamma distribution shape parameters, and substitution rates, were incorporated into MrBayes for each dataset. The SYM + I + G model was applied to the D2–D3 expansion segments of 28S rDNA, the GTR + I + G model to ITS1, and the TVM + I + G model to the partial *COI* gene. Each dataset was analyzed independently using four Markov chains over 10 × 10^6^ generations. Sampling occurred every 100 generations, with two independent runs per dataset. After discarding 30% of initial samples as burn-in and verifying convergence, the remaining samples were used to construct 50% majority-rule consensus trees. Posterior probabilities (PP) were calculated for all relevant clades, and trees were visualized using FigTree v1.4.4 (Rambaut, 2018).

## Results

Four *Longidorus* populations were recovered from downy oak forest in Thessaloniki (*n* = 2), Pieria (*n* = 1), and Tatoi (*n* = 1) (Table 1). Morphological and molecular analyses identified them as *L. aetnaeus*, *L. intermedius*, and *L. iranicus*, briefly described in this study. Likewise, four *Xenocriconemella* populations from Thessaloniki (*n* = 2), Pieria (*n* = 1), and Chalkidiki (*n* = 1) (Table 1) were identified as *X. iberica* Archidona-Yuste, Clavero-Camacho, Ruiz-Cuenca, Cantalapiedra-Navarrete, Liébanas, Castillo, and Palomares-Rius 2024 and *X. paraiberica* Archidona-Yuste, Clavero-Camacho, Ruiz-Cuenca, Cantalapiedra-Navarrete, Liébanas, Castillo, and Palomares-Rius, 2024. As type populations of both species have been molecularly characterized using the D2–D3 expansion segments of 28S rDNA and the ITS rDNA loci (Archidona-Yuste et al., 2024), no additional phylogenetic analyses were performed on the Greek specimens.

**Figure 1:**
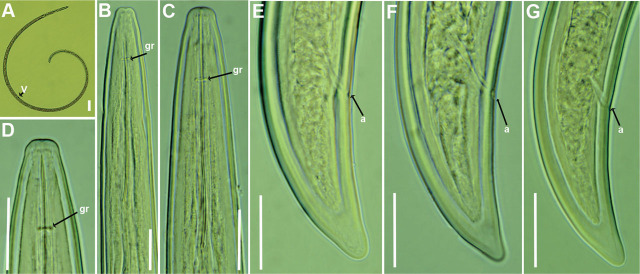
Light micrographs of *Longidorus aetnaeus* Roca, Lamberti, Agostinelli, and Vinciguerra, 1986, from Greece (A–G). (A) Whole female, (B–D) Female anterior regions, (E–G) Female tail regions. Abbreviations: a = anus; gr = guiding ring; V = vulva. Scale bars: A = 100 µm, B–G = 20 µm.

Greek population of *Longidorus aetnaeus* Roca, Lamberti, Agostinelli, and Vinciguerra, 1986, is shown in Figure 1.

### Measurements

The morphometric characterization of *L. aetnaeus*, *L. intermedius*, and *L. iranicus* is given in Table 2.

**Table 2:** Morphometric characterization of adult females of *Longidorus aetnaeus* Roca, Lamberti, Agostinelli and Vinciguerra, 1986, *Longidorus intermedius* Kozlowska and Seinhorst, 1979, and *Longidorus iranicus* Sturhan and Barooti, 1983 from Greece.

Trait^*^/locality	***Longidorus aetnaeus***	***Longidorus intermedius***		***Longidorus iranicus***
		
	**Thessaloniki**	**Thessaloniki**	**Pieria**	**Tatoi**
*n*	5	6	6	2
L	2.83 ± 0.28 (2.52–3.24)	3.88 ± 0.42 (3.23–4.39)	3.93 ± 0.39 (3.33–4.34)	(5.23, 5.61)
a	72.9 ± 5.9 (66.9–80.0)	76.8 ± 7.7 (64.6–86.1)	78.8 ± 6.5 (68.0–86.8)	(100.5, 105.9)
b	10.2 ± 0.9 (9.3–11.6)	9.9 ± 0.8 (8.8–10.8)	10.0 ± 0.8 (8.8–11.0)	(13.9, 16.3)
c	61.4 ± 3.0 (58.1–64.8)	93.6 ± 8.2 (78.8–101.8)	95.8 ± 8.7 (87.5–108.8)	(149.3, 155.9)
c′	2.0 ± 0.1 (1.8–2.1)	1.2 ± 0.1 (1.1–1.3)	1.2 ± 0.2 (1.1–1.4)	(0.9, 0.9)
d	2.6 ± 0.1 (2.6–2.7)	2.6 ± 0.1 (2.5–2.8)	2.6 ± 0.2 (2.5–2.8)	(3.0, 3.1)
d′	1.9 ± 0.1 (1.9–2.0)	2.1 ± 0.1 (1.9–2.3)	2.0 ± 0.1 (1.8–2.1)	(2.3, 2.3)
V	46.5 ± 1.5 (44.2–48.3)	47.5 ± 2.3 (44.7–51.1)	47.0 ± 1.2 (45.8–48.7)	(48.6, 49.0)
Odontostyle length	75.5 ± 1.6 (74.0–78.0)	115.1 ± 2.4 (112.5–119.0)	113.3 ± 5.6 (108.0–122.0)	(97.0, 104.0)
Odontophore length	41.6 ± 0.9 (41.0–43.0)	61.5 ± 1.0 (60.0–63.0)	60.7 ± 3.1 (55.0–63.0)	(51.0, 55.0)
Total stylet length	117.1 ± 2.5 (115.0–121.0)	176.6 ± 3.3 (173.5–182.0)	174.0 ± 7.0 (168.0–185.0)	(148.0, 159.0)
Anterior end to guide ring	23.8 ± 1.0 (23.0–25.5)	29.8 ± 1.6 (28.5–32.0)	30.6 ± 1.8 (27.5–32.5)	(30.0, 33.0)
Tail length	46.2 ± 4.8 (41.0–50.0)	41.6 ± 4.4 (37.5–48.0)	41.1 ± 3.1 (36.5–44.5)	(35.0, 36.0)
Hyaline part of tail length	14.3 ± 1.7 (12.0–16.0)	12.8 ± 0.7 (12.0–14.0)	13.3 ± 1.3 (11.5–15.0)	(12.0, 12.5)
**Width at level of:**				
lip region	9.2 ± 0.4 (9.0–10.0)	11.5 ± 0.3 (11.0–12.0)	11.6 ± 0.5 (11.0–12.0)	(10.0, 10.5)
vulva or mid-body	38.8 ± 2.0 (36.5–41.0)	50.6 ± 3.5 (45.5–56.0)	49.9 ± 4.0 (45.0–54.0)	(52.0, 53.0)
anus	23.5 ± 1.8 (21.0–25.5)	34.5 ± 3.0 (31.0–39.0)	33.5 ± 3.7 (30.0–39.0)	(38.0, 39.0)

All measurements are expressed in micrometers (μm) and presented as mean ± standard deviation (range), except for body length, which is given in millimeters (mm).

* Abbreviations are defined in Jairajpuri and Ahmad (1992).

### Description

*Female*: This population from Thessaloniki was characterized by a moderate female body length. The female habitus was ventrally curved, forming a close C-shape to a single spiral when killed by gentle heat, with pronounced curvature in the posterior half. Cuticle 2.5–3.0 µm thick at midbody. The lip region was conoid-rounded, either continuous with the body contour or slightly offset by a shallow depression, and anteriorly flattened. The amphidial pouch was asymmetrically bilobed. A single guiding ring was present, located 2.6–2.7 times the lip region diameter from the anterior end. Odontostyle well-developed and with slight basal muscular swellings, 1.8 times longer than the length of the odontophore. Pharynx extending to a terminal pharyngeal bulb 74.5 (70–77) µm long, with the dorsal gland nucleus (DN) and ventrosublateral gland nuclei (SVN) situated at approximately 33 (30–35)% and 51 (44–58)% of the distance from the anterior end of the pharyngeal bulb, respectively. Glandularium measuring 65.5 (62–69) µm. Cardia conoid-rounded. Vulva located near midbody or slightly anterior (44.2%–48.3%). Vagina 11 (9.5–13.0) µm in length, and the ovijector 23 (20–28) µm in width. Reproductive system amphidelphic, with equally developed anterior and posterior branches measuring 207–254 µm and 197–251 µm, respectively. Rectum 21 (20–23) µm long. Tail conoid, dorsally convex, ventrally concave with a bluntly rounded terminus.

*Male*: Not found.

*Juveniles*: Not found.

### Remarks

According to the polytomous key by Chen et al. (1997), and additional character states introduced by Peneva et al. (2013), the following codes correspond to the present population (with exceptions in parentheses): A2 – B1 – C2 – D3 – E3 – F1(2) – G1 – H5 – I1 – J1 – K?

This is the first report of this species from Greece. Aside from the original description from the rhizosphere of *Quercus ilex* L. in Sicily, Italy (Roca et al., 1986), the species has also been reported from Bulgaria, Georgia, Iran, Russia, and Serbia (Peneva and Nedelchev, 1995; Barsi and Lamberti, 2004; Amrei et al., 2013; Palomares-Rius et al., 2017). The morphology and morphometrics of the present population are consistent with those of the type and other previously reported populations, with minor differences observed in a ratio (66.9–80.0 *vs*. 77–91) and odontophore length (41–43 µm *vs*. 32–38 µm) (Table 1). These slight morphometric variations may reflect intraspecific geographical variability.

**Figure 2:**
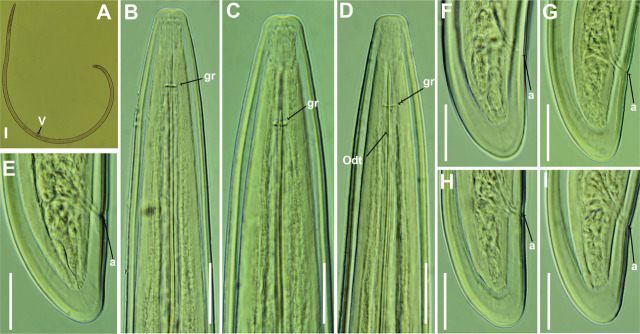
Light micrographs of *Longidorus intermedius* Kozlowska and Seinhorst, 1979 from Greece (A–I). (A) Whole female, (B–D) Female anterior regions, (E–O) Female tail regions. Abbreviations: a = anus; gr = guiding ring; V = vulva. Scale bars: A = 100 µm, B–O = 20 µm.

Greek population of *Longidorus intermedius* Kozlowska and Seinhorst, 1979, is shown in Figure 2.

### Measurements

The morphometric characterization of *L. aetnaeus*, *L. intermedius*, and *L. iranicus* is given in Table 2.

### Description

The populations from Thessaloniki and Pieria were characterized by a moderately long-bodied female. Female habitus ventrally curved, forming a close C-shape to a single open spiral when killed by gentle heat. Cuticle 2.5–3.0 µm thick at midbody. Lip region was rounded, flattened anteriorly, continuous with the body contour. The amphidial pouch is symmetrically bilobed. The guiding ring is located 2.5–2.8 times the lip region diameter from the anterior end. Odontostyle long, 1.7–2.1 times the length of the odontophore, which was well-developed and displayed slight basal swellings. Pharynx extending to a terminal pharyngeal bulb 103 (94–109) µm long with DN and SVN situated at approximately 29 (26–31)% and 49 (45–55)% of the distance from the anterior end of the pharyngeal bulb, respectively. Glandularium 91 (81–97) µm long. Vulva located near midbody or slightly anterior (45.8%–48.7%). Vagina 14.5 (13.0–15.5) µm in width, and the ovijector 26 (24–29) µm in width. Reproductive system amphidelphic, with equally developed anterior and posterior branches measuring 246–456 µm and 219–442 µm, respectively. Rectum 25 (23–27) µm long. Tail bluntly conoid with a widely rounded terminus.

*Male*: Not found.

*Juveniles*: Not found.

### Remarks

According to the polytomous key by Chen et al. (1997), and additional character states introduced by Peneva et al. (2013), the following codes correspond to the present populations (with exceptions in parentheses): A4 – B (1)2 – C2(3) – D13 – E2 – F2 –G1(2) – H2 – I1 – J1 – K5.

This is the first morphometric and molecular characterization of this species from Greece. It had previously been mentioned in an unpublished report by Peneva from Kavala, northern Greece, in association with *Quercus coccifera* L. (Tzortzakakis et al., 2008). The species has been recorded in various European countries, including Belgium, Bulgaria, the Czech Republic, Germany, Italy, Poland, the Netherlands, Iran, Russia, Slovakia, and Spain (Peneva et al., 2001; Rubtsova et al., 2001; Kumari et al., 2009; Subbotin et al., 2014; Archidona-Yuste et al., 2016; Monemi et al., 2024). The morphology and morphometrics of presently recovered populations closely match those of the type specimens and previously described populations (Kozlowska and Seinhorst, 1979; Peneva et al., 2001; Kumari et al., 2009; Subbotin et al., 2014; Archidona-Yuste et al., 2016).

### Measurements

The morphometric characterization of *L. aetnaeus*, *L. intermedius*, and *L. iranicus* is given in Table 2.

### Remarks

Since this species was recently characterized in detail, both morphometrically and molecularly, from grapevine and olive in Crete, Greece (Tzortzakakis et al., 2014), a full description is not provided in this suty. Instead, only the relevant measurements and molecular markers are presented (Tables 1 and 2). The morphology and morphometrics of the presently studied population of *L. iranicus* from Tatoi closely match those of the original description from Iran by Sturhan and Barooti (1983), as well as subsequent reports from Crete (Tzortzakakis et al., 2014), Italy, Serbia, and Slovenia (Roca and Lamberti, 1994; Roca, 2006; Širca and Urek, 2009). Minor differences in body and odontostyle length were observed, suggesting some intraspecific morphometric variability. Nevertheless, the molecular markers align well with those deposited in NCBI.

According to the polytomous key by Chen et al. (1997), the following codes correspond to the studied population: A34 – B1 – C23 – D1 – E3 – F3 – G2 – H1 – I1.

**Figure 3:**
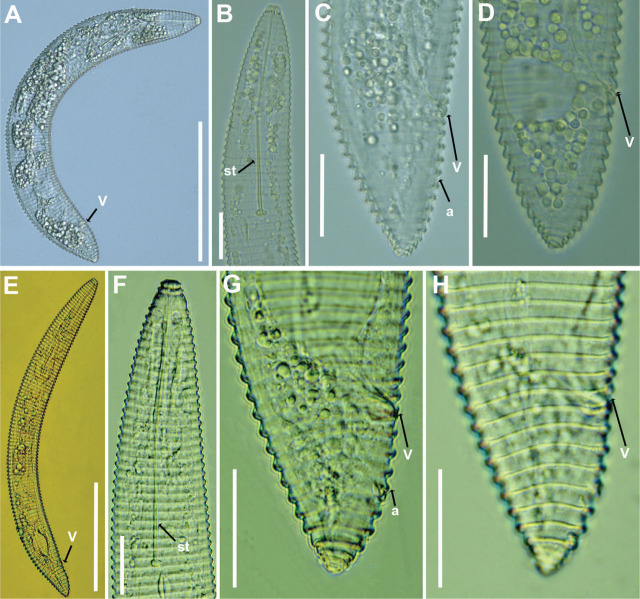
Light micrographs of *Xenocriconemella iberica* Archidona-Yuste, Clavero-Camacho, Ruiz-Cuenca, Cantalapiedra-Navarrete, Liébanas, Castillo, and Palomares-Rius 2024 (A–D) and *Xenocriconemella paraiberica* Archidona-Yuste, Clavero-Camacho, Ruiz-Cuenca, Cantalapiedra-Navarrete, Liébanas, Castillo, and Palomares-Rius 2024 (E–H) from Greece. (A, E) Whole female, (B, F) Female anterior regions, (C–H) Female tail regions. Abbreviations: a = anus; st = stylet; V = vulva. Scale bars: A, E = 100 µm, B–D, F–H = 20 µm.

Greek population of *Xenocriconemella iberica* Archidona-Yuste, Clavero-Camacho, Ruiz-Cuenca, Cantalapiedra-Navarrete, Liébanas, Castillo, and Palomares-Rius 2024 is shown in Figures 3A–D.

### Measurements

The morphometric characterization of *X. paraiberica* and *X. iberica* is given in Table 3.

**Table 3:** Morphometric characterization of adult females of *Xenocriconemella paraiberica* Archidona-Yuste, Clavero-Camacho, Ruiz-Cuenca, Cantalapiedra-Navarrete, Liébanas, Castillo, and Palomares-Rius, 2024 and *Xenocriconemella iberica* Archidona-Yuste, Clavero-Camacho, Ruiz-Cuenca, Cantalapiedra-Navarrete, Liébanas, Castillo, and Palomares-Rius, 2024 from Greece.

Trait^*^/locality	***Xenocriconemella paraiberica***			***Xenocriconemella iberica***
	
	**Thessaloniki**	**Pieria**	**Chalkidiki**	**Thessaloniki**
*n*	5	5	2	5
L	310.4 ± 41.1 (250–356)	304.2 ± 23.6 (270–333)	(274, 298)	309.8 ± 49.6 (248–354)
R	107.4 ± 5.9 (100–114)	105.6 ± 5.0 (100–111)	(102, 107)	104.6 ± 3.7 (100–110)
Rst	35.6 ± 2.6 (32–38)	33.4 ± 1.8 (31–36)	(33, 34)	35.2 ± 1.3 (34–37)
Roes	46.6 ± 1.8 (45–49)	45.2 ± 2.3 (43–49)	(44, 45)	44.8 ± 1.3 (43–46)
Rex	37.6 ± 2.5 (35–40)	35.8 ± 1.9 (34–39)	(35, 36)	37.4 ± 0.5 (37–38)
RV	11.8 ± 0.4 (11–12)	11.8 ± 0.8 (11–13)	(11, 12)	11.6 ± 0.5 (11–12)
Rvan	4.0 ± 0 (4–4)	3.8 ± 0.4 (3–4)	(4, 4)	4.0 ± 0 (4–4)
Ran	7.8 ± 0.4 (7–8)	8.0 ± 0.7 (7–9)	(7, 8)	7.6 ± 0.5 (7–8)
O	7.8 ± 0.3 (7.2–8.6)	7.9 ± 0.3 (7.5–8.2)	(7.7, 7.8)	8.0 ± 0.4 (7.4–8.4)
a	9.5 ± 1.2 (8.1–11.1)	9.8 ± 0.3 (9.3–10.1)	(9.4, 9.9)	9.4 ± 1.6 (7.1–11.4)
b	2.6 ± 0.3 (2.1–2.8)	2.5 ± 0.2 (2.2–2.7)	(2.2, 2.5)	2.7 ± 0.3 (2.3–3.0)
c	21.4 ± 2.9 (17.9–24.1)	22.6 ± 4.0 (18.7–28.9)	(18.6, 22.8)	21.2 ± 4.6 (15.5–25.7)
c′	0.7 ± 0.1 (0.7–0.8)	0.8 ± 0.1 (0.7–0.9)	(0.9, 0.9)	0.7 ± 0.1 (0.7–0.8)
V	90.2 ± 1.2 (88.5–91.6)	89.7 ± 1.8 (86.5–91.0)	(89.1, 89.9)	90.3 ± 0.4 (89.8–90.7)
VL/VB	1.0 ± 0.1 (0.8–1.1)	0.9 ± 0.2 (0.7–1.1)	(1.0, 1.1)	0.9 ± 0.1 (0.8–1.1)
Stylet	93.4 ± 3.6 (89.0–97.0)	91.0 ± 4.5 (86.0–97.0)	(91.0, 97.0)	93.0 ± 3.2 (88.0–96.0)
Pharynx	120.8 ± 4.9 (115–128)	122.4 ± 5.6 (117–131)	(118.0, 124.0)	114.6 ± 9.5 (102–126)
Maximum body width	32.8 ± 2.6 (30.0–36.0)	31.0 ± 1.6 (29.0–33.0)	(29.0, 30.0)	33.2 ± 4.1 (27.0–37.0)
Anal body width	19.8 ± 2.2 (17.5–23.5)	17.9 ± 3.7 (14.0–23.5)	(14.0, 18.0)	20.3 ± 2.9 (16.0–23.0)
Vulva to anus distance	13.4 ± 2.3 (11.0–17.0)	14.4 ± 1.9 (12.0–17.0)	(13.0, 14.0)	13.6 ± 2.1 (11.0–16.0)
Tail	14.5 ± 0.7 (14.0–15.5)	13.8 ± 2.6 (11.0–17.0)	(12.0, 16.0)	14.8 ± 1.2 (13.0–16.0

All measurements are expressed in micrometers (μm) and presented as mean ± standard deviation (range).

* Abbreviations are defined in Archidona-Yuste et al. (2024).

### Brief description

*Female*: Body ventrally arcuate, tapering slightly at both the anterior and posterior ends. Body annuli are smooth and lack anastomosis. The lip region consists of two annuli, not offset, and continuous with the body annuli. Stylet thin, long, and flexible, occupying 30.7 (26.3–37.1)% of body length, with slightly rounded knobs measuring 3.5–4.0 µm in width. Pharynx is typically criconematoid, with well-developed valves. Excretory pore located one to two annuli posterior to the level of the stylet knobs. The female genital tract is monodelphic, prodelphic, outstretched, and occupies 53.1 (49.2–61.7)% of body length. Anus situated 7.6 annuli (7–8) from the tail terminus. Tail conoid with a bluntly rounded terminus, and the annuli gradually decrease in both diameter and thickness toward the end.

### Remarks

The present population from Thessaloniki is morphologically and morphometrically consistent with the type population from Spain (Archidona-Yuste et al., 2024). It closely resembles both the type Spanish and the three Greek populations of *X. paraiberica* examined in this study. In fact, this population is extremely difficult to distinguish from *X. paraiberica* based on morphology and morphometry alone and can only be reliably differentiated using molecular markers.

Greek populations of *Xenocriconemella paraiberica* Archidona-Yuste, Clavero-Camacho, Ruiz-Cuenca, Cantalapiedra-Navarrete, Liébanas, Castillo, and Palomares-Rius 2024 is shown in Figures 3E–H.

### Measurements

The morphometric characterization of *X. paraiberica* and *X. iberica* is given in Table 3.

### Brief description

*Female*: Stylet thin, long, and flexible, occupying 26.9%–37.2% of the body length, with slightly rounded knobs measuring 3.5–4.0 µm in width. Pharynx is typically criconematoid, with well-developed valves. Excretory pore located one to three annuli posterior to the level of the stylet knobs. The female genital tract is monodelphic, prodelphic, and outstretched, occupying 47.3%–62.4% of the body length. Anus situated 7–9 annuli from the tail terminus. Tail conoid with a bluntly rounded terminus, and the annuli gradually decrease in diameter and thickness toward the end.

### Remarks

The three presently recovered populations of *X. paraiberica* from Thessaloniki, Pieria, and Chalkidiki are morphologically and morphometrically consistent with one another, as well as with the type population from Spain (Archidona-Yuste et al., 2024). They also closely resemble the type Spanish and presently studied Greek populations of *X. iberica*. In fact, these three populations are extremely difficult to distinguish from *X. iberica* based on morphology and morphometrics alone, and can only be reliably differentiated using molecular markers (see below).

### Molecular characterization of *Longidorus* spp. and *Xenocriconemella* spp. from Greece

Two *Longidorus* species from Greece (*L. aetnaeus* and *L. intermedius*) were molecularly characterized using sequences from two ribosomal regions, the D2–D3 expansion segments of the 28S rDNA and the ITS1 rDNA, as well as the mitochondrial *COI* gene. For *L. aetnaeus*, two sequences were obtained for the D2–D3 (705–713 bp; PV917559–PV917560), two for the ITS1 region (1,623–1,639 bp; PV891811–PV891812), and two for the *COI* gene (346 bp; PV871896–PV871897). No intraspecific variation was detected among ribosomal and mitochondrial sequences from *L. aetnaeus* specimens collected in Thessaloniki. For *L. intermedius*, 12 sequences were obtained for the D2–D3 region of the 28S rRNA (617–734 bp; PV917561–PV917572), 7 for the ITS1 region (1,532–1,551 bp; PV891813–PV891819), and 7 for the *COI* gene (344–346 bp; PV871898–PV871905). Low intraspecific variation was observed among D2–D3 expansion segments of the 28S rDNA sequences from *L. intermedius* specimens collected in Thessaloniki and Pieria (99.7%–99.8% identity), differing by 1–3 bp and showing no indels. Similarly, ITS1 sequences displayed high identity (99.2%–99.9%), differing by 2–12 bp with 0–1 indels. By contrast, no intraspecific variation was observed in the *COI* sequences within either the Thessaloniki or Pieria populations (100% identity). However, marked interpopulation divergence was detected between these localities, with only 86.0% sequence identity and a difference of 46 base pairs and 0 indels.

The expansion segments of the 28S rRNA sequences from *L. aetnaeus* in Thessaloniki were highly identical (98.5%–99.1%) to those available in NCBI for specimens from Russia, Ukraine, the USA, Georgia, and Iran (KF242318–KF242324, KF292307, KC357770–KC357771), differing by only 6–10 bp and 3–7 indels (Amrei et al., 2013; Subbotin et al., 2014; Poiras et al., data unpublished). These sequences also showed high identity (97.9%–98.3%) to those of *L. leptocephalus* from Russia, Greece, the UK, and Slovenia (KF242325–KF242327, ON241755–ON241758, AY601580, DQ364600), differing by 12–15 bp and 4 indels (He et al., 2005; Širca et al., 2007; Subbotin et al., 2014; Clavero-Camacho et al., 2022). By contrast, they were clearly distinct from all other *Longidorus* species in the NCBI database, the closest being *L. sabalanicus* Asgari, Eskandari, Castillo, and Palomares-Rius, 2022 from Iran (MZ474667), with 92.4% identity and differences of 54 bp and 7 indels (Asgari et al., 2022). The ITS1 sequence of *L. aetnaeus* from Greece (PV891811–PV891812), newly deposited in NCBI, showed 90.7% identity and low coverage (77%) compared to the ITS1 of *L. leptocephalus* (ON241759) from Greece, differing by 150 bp and 68 indels (Clavero-Camacho et al., 2022). It also differed markedly from all other *Longidorus* sequences available in NCBI, the closest being *L. piceicola* from Romania (LT669802–LT669803), with 88.2% identity and very low coverage (39%), differing by 77 bp and 44 indels (Groza et al., 2017). Finally, the two mitochondrial *COI* sequences of *L. aetnaeus* (PV871896–PV871897) displayed only partial identity to those of *L. aetnaeus* specimens from Russia (KY816656–KY816659), differing by 29–30 bp and no indels, with a sequence identity of 88.4%–88.8% (Palomares-Rius et al., 2017); and <85% identity to all other *Longidorus* spp. sequences deposited in NCBI, differing by >40 bp and 0–6 indels.

The D2–D3 sequences of *L. intermedius* from Thessaloniki and Pieria exhibited a high degree of identity (98.6%–100%) to those available in GenBank for specimens from Germany, Russia, and Spain (AF480074, KF242311–KF242312, K T308868, JX445117), differing by only 0–10 base pairs (bp) and 0–5 insertions/deletions (indels) (Rubtsova et al., 2001; Gutiérrez-Gutiérrez et al., 2013; Subbotin et al., 2014; Archidona-Yuste et al., 2016). These sequences also showed high identity (99.7%) to those of *L. piceicola* from Romania and Slovakia (LT669801, AY601577, KY086070), differing by just 2 bp and no indels (He et al., 2005; Groza et al., 2017). By contrast, the identity value was lower (97.2%–97.4%) than that of *L. uroshis* and *L. carpathicus* from Slovakia and Germany (EF538754 and AF480072, respectively), differing by 21 bp and 3 indels (Rubtsova et al., 2001; Kumari et al., 2009).

The ITS1 sequences of *L. intermedius* from Thessaloniki and Pieria exhibited high identity (97.8%–98.8%) to those available in NCBI for *L. piceicola* from Romania and *L. intermedius* specimens from Spain (LT669802–LT669803, KT308890), differing by only 20–22 bp and 5–8 indels (Archidona-Yuste et al., 2016; Groza et al., 2017). By contrast, these sequences showed <87% identity to all other *Longidorus* spp. sequences deposited in NCBI, differing by >125 bp and 43 indels.

Due to the substantial divergence between the mitochondrial *COI* sequences of *L. intermedius* populations from Thessaloniki and Pieria (PV871902–PV871905, 86.2% identity, differing by 46 bp and 0 indels in each population), both sets of sequences were analyzed independently in comparison with other *Longidorus* *COI* sequences available in NCBI. The *COI* sequences from *L. intermedius* in Thessaloniki (PV871898–PV871901) exhibited only partial identity (78.9%–80.1%) to four *Longidorus* species – *L. poessneckensis*, *L. maginicus* Liébanas, Clavero-Camacho, Cantalapiedra-Navarrete, Guerrero, Palomares-Rius, Castillo, and Archidona-Yuste, 2022, *L. iranicus*, and *L. oakgracilis* Cai, Archidona-Yuste, Cantalapiedra-Navarrete, Palomares-Rius and Castillo, 2020, originating from Slovakia, Spain, Greece, and Spain, respectively (KY816595, OL471046, KY816677, MK937586), differing by 63–71 bp and 0–2 indels (Kumari et al., 2009; Palomares-Rius et al., 2017; Cai et al., 2020; Liébanas et al., 2022). Similarly, the *COI* sequences from *L. intermedius* in Pieria (PV871902–PV871905) showed only moderated identity (77.0%–83.3%) to three *Longidorus* species, *L. intermedius*, *L. macrosoma*, and *L. helveticus*, from Spain, Austria, and the Czech Republic, respectively (KY816676, EF538746, JN627416), differing by 42–76 bp and 0–6 indels (Kumari et al., 2009; Kumari and Subbotin, 2012; Palomares-Rius et al., 2017).

*Xenocriconemella* spp. from Greece (*X. paraiberica* and *X. iberica*) were molecularly characterized using sequences from two ribosomal regions: the D2–D3 expansion segments of the 28S rDNA and the ITS rDNA. For each population, five D2–D3 sequences were obtained (*X. paraiberica*: 607–674 bp, PV917579–PV917590; *X. iberica*: 650–669 bp, PV917574–PV917578), along with five ITS rDNA sequences (*X. paraiberica*: 735–830 bp, PV891825–PV891836; *X. iberica*: 630–683 bp, PV891820–PV891824). The only exception was the Chalkidiki population, for which two sequences per marker were obtained (*X. paraiberica* D2–D3: 666–673 bp, PV917589–PV9175890; ITS: 796 bp, PV891835–PV891836). No intraspecific variation was observed in the 28S D2–D3 or ITS sequences of *X. paraiberica* across localities, Thessaloniki (PV917579–PV917583, PV891825–PV891829), Pieria (PV917584–PV917588, PV891830–PV891834), and Chalkidiki (PV891889–PV891890, PV891835–PV891836), with 100% sequence identity. By contrast, *X. iberica*, represented by a single population from Thessaloniki (PV917574–PV917578, PV891820–PV891824), exhibited low intraspecific variation for D2–D3 and ITS (99.5%–100.0%, 98.7%–99.0%, respectively), differing by 0–3 bp, 0 indels, and 7–8 bp, 0–1 indels, respectively.

D2–D3 sequences from *X. paraiberica* collected in Thessaloniki, Pieria, and Chalkidiki were highly identical (99.1%–99.6%) to those from the original Spanish populations (OR880152–OR880167), differing by only 3–6 bp and 0–2 indels (Archidona-Yuste et al., 2024). These sequences also showed the following identities to those of other *Xenocriconemella* species: 95.1%–95.4% with those of *X. andreae* Cantalapiedra-Navarrete, Clavero-Camacho, Criado-Navarro, Salazar-García, García-Velázquez, Palomares-Rius, Castillo, and Archidona-Yuste, 2024 from Spain and Portugal (31–32 bp differences, 0–2 indels; PP833567–PP833574; Cantalapiedra-Navarrete et al., 2024); 94.4%–94.5% with those of *X. tica* Peraza-Padilla, Artavia-Carmona, Aráuz-Badilla, Liébanas, Cantalapiedra-Navarrete, Salazar-García, García-Velázquez, Palomares-Rius, Castillo, and Archidona-Yuste, 2025 from Costa Rica (37 bp, 0 indels; PV435862–PV435866; Peraza-Padilla et al., 2025); 94.1%–94.4% with those of *X. costaricense* Peraza-Padilla, Aráuz-Badilla, Cantalapiedra-Navarrete, Palomares-Rius, Archidona-Yuste, and Castillo, 2024 (37–40 bp, 0 indels; PP209388–PP209391; Peraza-Padilla et al., 2024); 91.9%–92.2% with those of *X. iberica* from Spain (53–54 bp, 3–5 indels; OR880107–OR880145; Archidona-Yuste et al., 2024); 91.9% with those of *X. iberica* from Thessaloniki (53–54 bp, 3 indels; PV917574–PV917578; this study); and 91.3%–91.4% with those of *X. pradense* Archidona-Yuste, Clavero-Camacho, Ruiz-Cuenca, Cantalapiedra-Navarrete, Liébanas, Castillo, and Palomares-Rius, 2024 from Spain (58 bp, 3 indels; OR880209–OR880217; Archidona-Yuste et al., 2024). Similarly, sequences of *X. iberica* from Thessaloniki were highly identical (99.4%–99.6%) to those from Spain (OR880107–OR880145), differing by 3–4 bp and no indels (Archidona-Yuste et al., 2024). These also showed the following identities to those of other *Xenocriconemella* species: 92.5%–93.4% with those of *X. andreae* (44–50 bp, 4–9 indels; PP833567–PP833574; Cantalapiedra-Navarrete et al., 2024); 91.3%–91.8% with those of *X. paraiberica* (55–57 bp, 3–5 indels; OR880152–OR880200; Archidona-Yuste et al., 2024); 90.5%–90.7% with those of *X. costaricense* (63–64 bp, no indels; PP209388–PP209391; Peraza-Padilla et al., 2024); and 90.4% with those of *X. tica* (65 bp, 11 indels; PV435862–PV435866; Peraza-Padilla et al., 2025).

ITS sequences of *X. paraiberica* collected in Thessaloniki, Pieria, and Chalkidiki were identical (95.0%–96.0%) to those from the original Spanish populations (OR878338–OR878349), differing by 29–42 bp and 22–31 indels (Archidona-Yuste et al., 2024). These sequences were clearly distinct from those of all other *Xenocriconemella* species (<90% identity) and exhibited low coverage.

### Phylogenetic analysis

**Figure 4:**
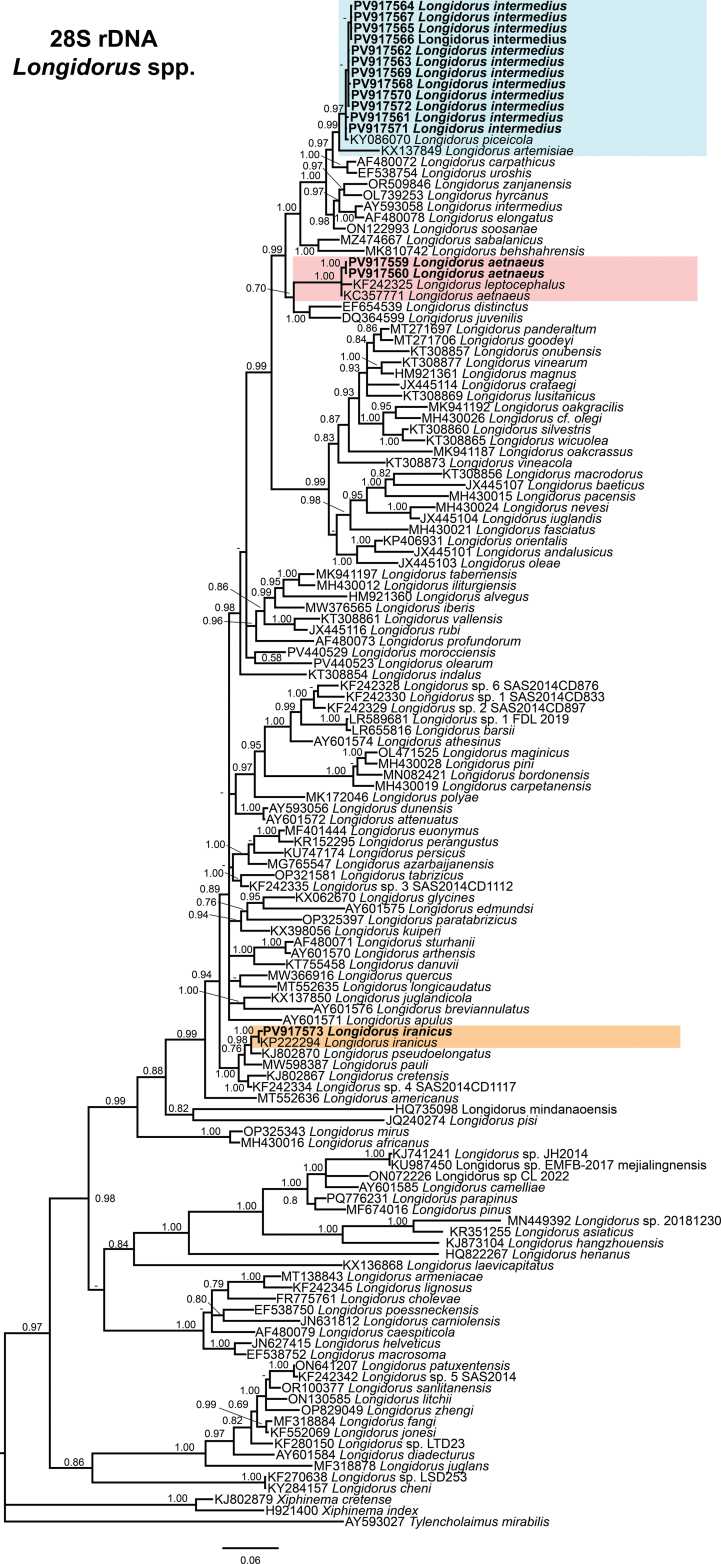
Phylogenetic relationships of *Longidorus aetnaeus* Roca, Lamberti, Agostinelli, and Vinciguerra, 1986 and *Longidorus intermedius* Kozlowska and Seinhorst, 1979 from Greece within *Longidorus* spp., inferred from a Bayesian 50% majority-rule consensus tree based on D2–D3 expansion segments of the 28S rDNA gene. The analysis was conducted under the symmetrical model with invariable sites and gamma distribution (SYM + I + G): − lnL = 17367.5440; AIC = 35293.087920; freqA = 0.2500; freqC = 0.2500; freqG = 0.2500; freqT = 0.2500; R(a) = 0.6301; R(b) = 2.3716; R(c) = 1.2643; R(d) = 0.3973; R(e) = 4.2679; R(f) = 1.0000; Pinva = 0.2970; and Shape = 0.7080. PP >0.70 are indicated at the relevant nodes. Newly generated sequences are shown in bold. The scale bar represents the expected number of substitutions per site. Colored boxes denote clade associations of *Longidorus* species included in this study. AIC, Akaike information criterion; PP, posterior probabilities.

**Figure 5:**
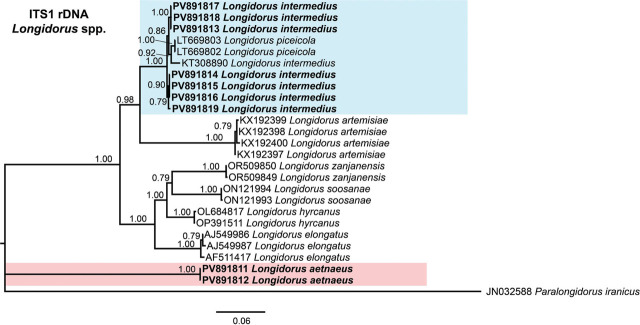
Phylogenetic relationships of *Longidorus aetnaeus* Roca, Lamberti, Agostinelli, and Vinciguerra, 1986 and *Longidorus intermedius* Kozlowska and Seinhorst, 1979 from Greece within *Longidorus* spp., inferred from a Bayesian 50% majority-rule consensus tree based on ITS1 rDNA gene. The analysis was conducted under the general time-reversible model and a gamma distribution (GTR + G): −lnL = 6986.5651; AIC = 14091.130240; freqA = 0.2493; freqC = 0.2071; freqG = 0.2721; freqT = 0.2715; R(a) = 0.7154; R(b) = 2.1386; R(c) = 0.7569; R(d) = 0.3744; R(e) = 3.2444; R(f) = 1.0000; and Shape = 0.7090. PP >0.70 are indicated at the relevant nodes. Newly generated sequences are shown in bold. The scale bar represents the expected number of substitutions per site. Colored boxes denote clade associations of *Longidorus* species included in this study. AIC, Akaike information criterion; ITS, internal transcribed spacer; PP, posterior probabilities.

**Figure 6:**
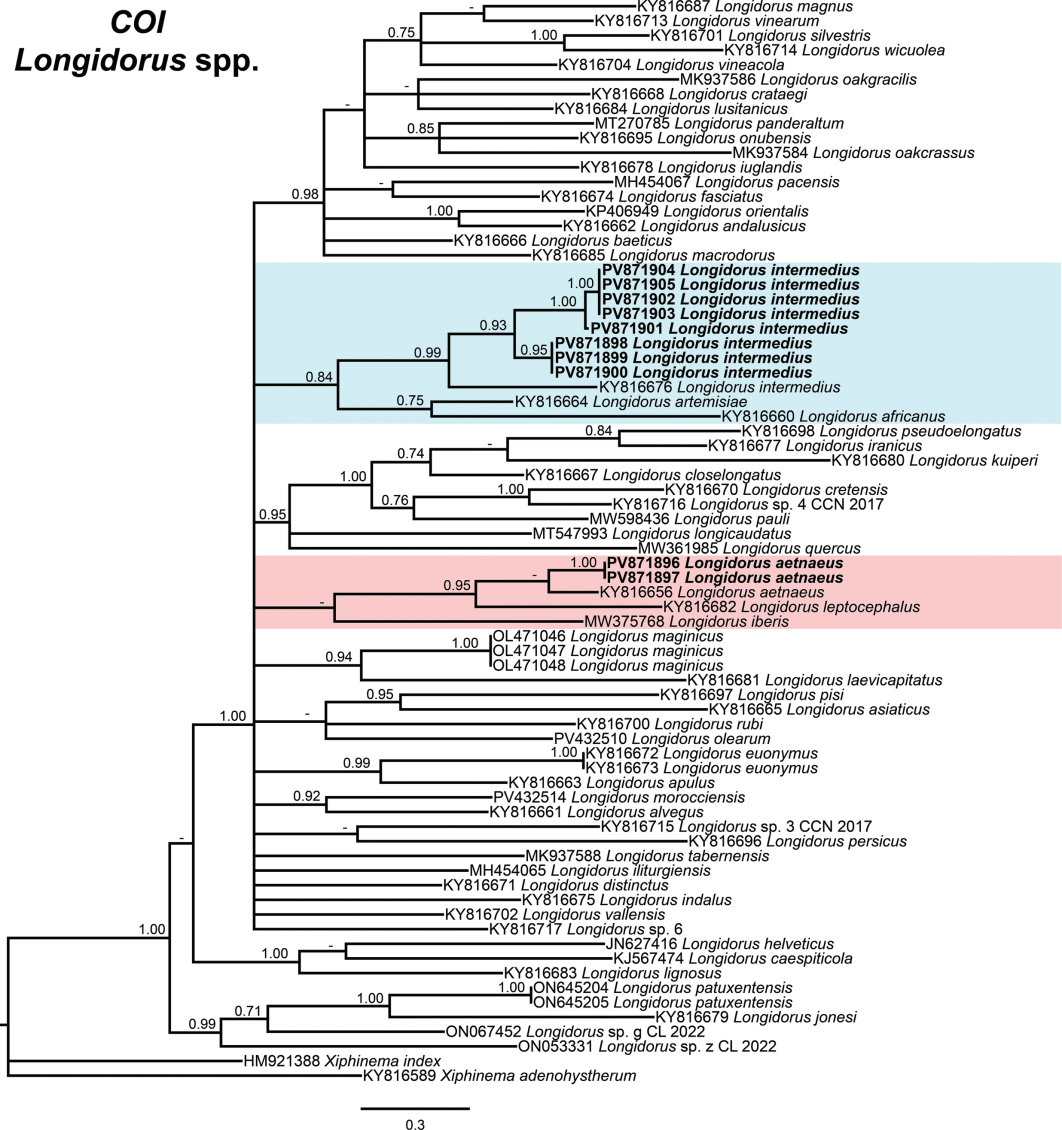
Phylogenetic relationships of *Longidorus aetnaeus* Roca, Lamberti, Agostinelli and Vinciguerra, 1986 and *Longidorus intermedius* Kozlowska and Seinhorst, 1979 from Greece within *Longidorus* spp., inferred from a Bayesian 50% majority-rule consensus tree based on *COI* mtDNA gene. The analysis was conducted under the transversion model with invariable sites and a gamma-shaped distribution (TVM + I + G): −lnL = 11077.4604; AIC = 22464.920820; freqA = 0.2466; freqC = 0.2026; freqG = 01391; freqT = 0.4116; R(a) = 0.4113; R(b) = 13.4611; R(c) = 0.9401; R(d) = 2.0351; R(e) = 13.4611; R(f) = 1.0000; Pinva = 0.3360; and Shape = 0.3420. PP >0.70 are indicated at the relevant nodes. Newly generated sequences are shown in bold. The scale bar represents the expected number of substitutions per site. Colored boxes denote clade associations of *Longidorus* species included in this study. AIC, Akaike information criterion; PP, posterior probabilities.

Phylogenetic analyses of *Longidorus* species were conducted using BI based on the D2–D3 expansion segments of 28S rDNA, ITS1 rDNA, and partial *COI* mtDNA sequences (Figs. 4–6, respectively). The resulting phylogenetic trees, reconstructed from ribosomal and mitochondrial DNA markers, included 137, 26, and 74 sequences, with alignments comprising 765, 1,582, and 401 characters, respectively. The Bayesian 50% majority-rule consensus tree inferred from the D2–D3 expansion segments of 28S rDNA is presented in Figure 4.

In the D2–D3 expansion segments of 28S rRNA phylogeny, the sequences of *L. intermedius* and *L. aetnaeus* formed a well-supported clade (PP = 0.99) with other sequences of *Longidorus* species, including the sequence of *L. piceicola* Lisková, Robbins and Brown, 1997 from Romania (KY086070), *L. artemisiae* Rubtsova, Chizhov and Subbotin, 1999 from Poland (KX137849), the sequence of *L. carpathicus* Lisková, Robbins and Brown, 1997 from Germany (AF480072), the sequence of *L. uroshis* Krnjaic, Lamberti, Krnjaic, Agostinelli and Radicci, 2000 from Slovakia (EF538754), the sequence of *L. zanjanensis* Asgari, Eskandari, Castillo, and Palomares-Rius, 2023 from Iran (OR509846), the sequence of *L. hyrcanus* Mobasseri, Pourjam, Farashiani and Pedram, 2023 from Iran (OL739253), the sequence of *L. intermedius* from the Netherlands (AY593058), the sequence of *L. elongatus* (de Man, 1876) Micoletzky, 1922 from Belgium (AF480078), the sequence of *L. soosanae* Ehtesham, Pedram, Atighi and Jahanshahi, 2023 from Iran (ON122993), the sequence of *L. sabalanicus* from Iran (MZ474667), the sequence of *L. behshahrensis* Bakhshi Amrei, Peneva, Rakhshandehroo, and Pedram, 2020 from Iran (MK810742), the sequence of *L. distinctus* Lamberti, Choleva and Agostinelli, 1983 from Slovakia (EF654539), and the sequence of *L. juvenilis* Dalmasso, 1969 from Slovenia (DQ364599) (Fig. 4). By contrast, the sequence of *L. iranicus* formed a separate subclade, clustering with the sequence of *L. pseudoelongatus* from Greece (KJ802870) and a sequence of *L. iranicus* (KP222294).

In the ITS region phylogenetic tree (Fig. 5), the analysis revealed that the sequences of *L. intermedius* (PV891813–PV891819) formed a well-supported subclade (PP = 1.00) with sequences of *L. piceicola* from Romania (LT669802–LT669803) and the sequence of *L. intermedius* from Spain (KT308890). By contrast, the sequences of the population of *L. aetnaeus* (PV891811–PV891812) exhibited substantial divergence from all other sequences of *Longidorus* species, clustering separately as an outgroup (Fig. 5).

Although phylogenetic relationships based on the *COI* gene were not well-resolved, the sequences of *L. intermedius* (PV871898–PV871905) clustered together in a distinct, moderately supported clade (PP = 0.84), along with the sequence of *L. intermedius* from Russia (KY816676), the sequence of *L. artemisiae* from Russia (KY816664), and the sequence of *L. africanus* from Tunisia (KY816660). By contrast, the sequence of *L. aetnaeus* (PV871896–PV871897) exhibited substantial divergence from the sequence of a Russian population of *L. aetnaeus* (KY816656), forming a moderately supported subclade (PP = 0.95) with the sequence of *L. leptocephalus* from Russia (KY816682) (Fig. 6).

## Discussion

Nematode populations from forested areas in Central and Northern Greece were identified as five species: *X. iberica*, *X. paraiberica*, *L. aetnaeus*, *L. intermedius*, and *L. iranicus*, based on integrative taxonomy and phylogenetic analyses using nuclear rDNA and mitochondrial DNA markers. This study provides the first record of *X. iberica* and *X. paraiberica* in Greece and the first report of these species outside the Iberian Peninsula (Archidona-Yuste et al., 2024), expanding the known distribution of *Xenocriconemella* in the Mediterranean Basin. These findings underscore the need for continued surveys in natural habitats to uncover the global biodiversity of the genus (Archidona-Yuste et al., 2024).

The use of ribosomal (D2–D3, ITS1) and mitochondrial (*COI*) markers proved effective for species identification and revealed new diversity within *Longidorus* in Greece. Molecular data confirmed that *L. intermedius* and *L. piceicola*, although differing morphologically in lip region width (11–12 µm vs. 14–17 µm), odontostyle length [111 (105–118) µm *vs* 160 (151–169) µm], and body size [4.1 (3.6–4.5) mm *vs* 5.2 (4.2–6.0) mm], share high ribosomal sequence identity (Groza et al., 2017). This suggests a possible recent speciation event, warranting further study using additional nuclear (e.g., *hsp90*) and mitochondrial markers. Notably, *COI* analysis revealed high genetic divergence (up to 15.5%) between *L. intermedius* populations from Thessaloniki and Pieria, comparable to intraspecific variation in *L. orientalis* (Subbotin et al., 2015).

Phylogenetic trees based on D2–D3, ITS1, and *COI* markers aligned with previous studies (Gutiérrez-Gutiérrez et al., 2013; Archidona-Yuste et al., 2019; Amrei et al., 2020; Cai et al., 2020; Inserra et al., 2020; Clavero-Camacho et al., 2021), supporting the morphological identification of *L. aetnaeus*, *L. intermedius*, and *L. iranicus*. This research increased the number of *Longidorus* species in Greece and revealed the molecular diversity within *Longidorus*. These species clustered with others sharing rounded lip regions, moderate odontostyle lengths, and conoid-rounded tails, reinforcing the congruence between molecular and morphological traits.

In conclusion, this study increases the prodigious biodiversity of *Xenocriconemella* and *Longidorus* in Greece by adding new species records.
